# Evaluation of a Method for Issuing Warnings Pre-epidemics and Epidemics in Japan by Infectious Diseases Surveillance

**DOI:** 10.2188/jea.14.33

**Published:** 2005-03-18

**Authors:** Yoshitaka Murakami, Shuji Hashimoto, Kiyosu Taniguchi, Ken Osaka, Hiroshi Fuchigami, Masaki Nagai

**Affiliations:** 1 Epidemiology and International Health Research Section, Environmental Health Sciences Division, National Institute for Environmental Studies.; 2 Department of Hygiene, Fujita Health University School of Medicine.; 3 Infectious Disease Surveillance Center, National Institute of Infectious Diseases.; 4 Saitama Health Promotion Corporation.; 5 Department of Public Health, Saitama Medical School.

**Keywords:** communicable disease, surveillance, disease outbreaks, warning, evaluation studies

## Abstract

BACKGROUND: Simple methods have been developed to warn of pre-epidemics and epidemics in small areas using data of infectious diseases surveillance. Epidemic warnings are made if the index of cases per week per sentinel medical institution is greater than a defined value. A pre-epidemic warning means that an epidemic warning will be given in the following four weeks. While the methods are used routinely for surveillance in Japan, they remain to be validated.

METHODS: Infectious diseases surveillance data of influenza-like illness and 12 pediatric diseases in the fiscal year between 1999 and 2001 were used in the analysis. We examined the frequency of warnings, temporal changes in the index before and after the onset of a warning, and the sensitivity, specificity, and positive predictive value of pre-epidemic warnings.

RESULTS: For the majority of the diseases investigated, the proportion of weeks in which a warning was issued ranged between 0% and 10%. In several diseases including influenza-like illness, we observed a rapid increase and gradual decrease in the index before and after a warning. The sensitivity, specificity, and positive predictive value of a pre-epidemic warning were 90.4%, 93.7% and 23.9% for influenza-like illness, and ranged between 25.1-54.2%, 86.1-99.2%, and 2.5-20.8% for the pediatric diseases (chickenpox, rubella, measles, and mumps), respectively.

CONCLUSIONS: The study showed that the methods used for determining whether or not to issue an epidemic warning were satisfactory in some diseases, including influenza-like illness, and may need to be improved in several other diseases.

The surveillance of infectious diseases is conducted in many countries.^1^^-^^9^ Numerous methods for detecting and forecasting epidemics from the surveillance data have been developed and evaluated, with some of these methods being used in surveillance systems.

A surveillance system of infectious diseases has been introduced in Japan with the aim of detecting epidemics in small areas (e.g. a public health center area).^9^ The application of a warning system for small areas is unique to Japan when compared with other countries. The requirement for pre-epidemic and epidemic warnings of several infectious diseases are reviewed weekly in every public health center area using surveillance data. The information on influenza-like illness is then made available to the general public through the World Wide Web (http://idsc.nih.go.jp/others/topics/inf-keiho/trend02.html, Accessed on February 10, 2004). In April 1999, the surveillance system was expanded by the Infectious Disease Control Law. Prior to this law change, warnings of influenza-like illness were based on reports on the number of cases each week in sentinel medical institutions, selected mainly from pediatric departments in hospitals and clinics. After this law change, the surveillance system was expanded to include sentinel medical institutions in internal medicine departments in hospitals and clinics. The criteria for determining whether to issue pre-epidemic and epidemic warnings for infectious diseases including influenza-like illness were developed using surveillance data collected prior to the law change. However, these original criteria have been evaluated on data acquired by the current surveillance system. We therefore carried out a study to evaluate the performance of these methods using surveillance data collected in Japan from April 1999 through March 2002.

## METHODS

### Surveillance of Infectious diseases in Japan

The National Epidemiological Surveillance of Infectious Diseases in Japan is organized by the Ministry of Health, Labor, and Welfare^9^ and is operated by the Infectious Disease Surveillance Center, National Institute of Infectious Diseases. The system collects data on influenza-like illness and pediatric diseases. Prior to the introduction of the Infectious Disease Control Law in April 1999, the sentinel medical institutions in the two surveillance systems were selected mainly from pediatric departments in hospitals and clinics. Following the introduction of this law, the influenza-like illness surveillance system also includes sentinel medical institutions of the internal medicine departments in hospitals and clinics. The number of sentinel medical institutions in April 2002 was 4,656 in the influenza-like illness surveillance system and 3,011 in the pediatric diseases surveillance system.^10^ The number of sentinel medical institutions in the health center areas was approximately proportional to their population size.^11^ Sentinel medical institutions were recruited on a voluntary basis, and each of the sentinel medical institutions sent weekly reports of the number of cases of notifiable diseases to the public health center. The public health center notified local government using an online computer network. The notifiable diseases were influenza-like illness in the influenza-like illness surveillance system and 12 diseases in the pediatric diseases surveillance system (Table 1).

**Table 1.** Critical values (cases per week per sentinel medical institution) for issuing (onset) and ending epidemic warnings, and issuing pre-epidemic warning in infectious disease surveillance in Japan.

Diseases	Warning		Pre-epidemic warning

	onset	end	
Influenza-like illness	30	10	10
Pharyngoconjunctival fever	1	0.1	—
Group A streptococcal pharyngitis	4	2	—
Infectious gastroenteritis	20	12	—
Chickenpox	7	4	4
Hand-foot-mouth disease	5	2	—
Erythema infectiosum	2	1	—
Exanthema subtium	4	2	—
Pertussis	1	0.1	—
Rubella	3	1	1
Herpangina	6	2	—
Measles	1.5	0.5	0.5
Mumps	5	2	3

The numbers in the table are cases per week per sentinel medical institution.

### The method for detecting epidemics

The warnings were based on an index calculated from the number of cases per week per sentinel medical institution. An epidemic warning in a public health center area was given if the index in the area exceeded the critical value for the onset of an epidemic and continued until the index in that area was lower than the critical value for the end of an epidemic.^12^ The critical value for the onset of an epidemic fell between the 95th and 99th percentiles of the distribution of indices in 1993-1997, while the critical values for the end of epidemic were defined as the 90th percentiles of the distribution.^13^ The method for issuing an epidemic warning was applied to data on influenza-like illness and 12 pediatric diseases. Like infectious disease surveillance in Japan, we made a pre-epidemic warning, which was issued before an epidemic warning, for five diseases (influenza-like illness, chickenpox, rubella, measles and mumps). These diseases would need much attention against early detection of epidemic for the public health activities. In a public health center area, a pre-epidemic warning was made only if the index in the area exceeded the critical value for a pre-epidemic warning. This meant that an epidemic warning would then be issued in that area in the following four weeks. The critical value was determined according to the sensitivity, specificity, and positive predictive value of a pre-epidemic warning calculated using surveillance data of 1993-1997. This method for determining a pre-epidemic warning was then applied to data on influenza-like illness and four pediatric diseases. Table 1 shows the critical values for each disease.

### Evaluation of the method for detecting epidemics

We analyzed surveillance data of infectious diseases collected from April 1999 through March 2002. The public health center areas in the survey in April 1999 (583 areas) were used for the analysis. The data set consists of reports on the number of cases per week per sentinel medical institution in each public health center area(583 areas) collected over the 3 fiscal years of the study (i.e.157 weeks).

The proportion of weeks, in which epidemic and pre-epidemic warnings was issued in the 583 public health center areas during the fiscal year of 1999-2001, was calculated and compared with similar data of 1993-1997. Temporal changes in the indices before and after an epidemic warning during 1999 and 2001 were examined for the evaluation of warning. The sensitivity, specificity, and positive predictive values of pre-warning were calculated for the evaluation of pre-warning. Sensitivity was calculated as the proportion of valid pre-warnings made four weeks prior to an epidemic warning, while specificity was calculated as the proportion of weeks in which no pre-epidemic warning was issued relative to the total number of weeks without an epidemic warning including the four weeks before and after the warning. The positive predictive value was defined as the proportion of valid pre-epidemic warnings relative to the total number of pre-epidemic warnings.

## RESULTS

The annual number of cases per sentinel medical institution is shown in Table 2. For many diseases, including influenza-like illness, the numbers of cases per sentinel medical institution in the fiscal years of 1999-2001 varied within or near the range of those recorded of 1993-1997. For group A streptococcal pharyngitis, chickenpox, exanthema subtium, and herpangina, the number of cases per sentinel medical institution was higher in the years 1999-2001 compared to 1993-1997. In contrast, the number of cases of rubella per sentinel medical institution was lower in the years 1999-2001 than in 1993-1997.

**Table 2.** Annual numbers of cases per sentinel medical institution in infectious disease surveillance in Japan, April 1999-March 2002, compared with range of those in 1993-1997.

Annual numbers of cases per sentinel medical institution					

Diseases	April 1999-March 2000	April 2000-March 2001	April 2001-March 2002	Range(1993-1997)	

				minimum	maximum
Influenza-like illness	193.13	57.01	144.50	44.75	312.12
Pharyngoconjunctival fever	4.12	7.85	7.76	1.76	4.39
Group A streptococcal pharyngitis	43.81	57.60	50.82	24.71	34.88
Infectious gastroenteritis	294.63	299.58	287.90	192.51	498.41
Chickenpox	84.02	97.85	83.54	73.40	77.98
Hand-foot-mouth disease	18.68	70.25	41.10	10.07	65.35
Erythema infectiosum	8.64	14.31	23.09	5.29	22.74
Exanthema subtium	42.79	42.67	40.70	34.22	36.75
Pertussis	1.16	1.18	0.56	1.14	2.34
Rubella	1.24	1.05	0.89	6.70	61.20
Herpangina	53.80	49.12	47.06	29.98	39.60
Measles	3.40	9.42	9.28	6.50	14.30
Mumps	30.89	58.13	83.66	29.21	62.37

Data of fiscal years are used in 3 years from 1999 through 2001.

Table 3 shows the proportions of weeks in which an epidemic warning for influenza-like illness or the 12 pediatric diseases was issued in the 583 public health center areas during the fiscal year of 1999-2001. These proportions were similar to those recorded in 1993-1997. In exanthema subtium, pertussis, and rubella, the proportions of weeks with an epidemic warning were lower in the years of 1999-2001 than in 1993-1997.

**Table 3.** The proportion of weeks in which an epidemic warnings were issued in public health center areas in April 1999-March 2002, compared with range of those in 1993-1997.

The proportion of epidemic warning weeks (%)					

Diseases	April 1999-March 2000	April 2000-March 2001	April 2001-March 2002	Range(1993-1997)	

				minimum	maximum
Influenza-like illness	3.3	0.5	5.5	0.7	10.9
Pharyngoconjunctival fever	3.2	6.4	7.3	1.8	5.0
Group A streptococcal pharyngitis	4.6	7.7	6.4	3.6	5.2
Infectious gastroenteritis	6.5	6.9	6.0	3.6	7.3
Chickenpox	2.8	4.3	2.7	4.1	4.7
Hand-foot-mouth disease	1.7	10.5	5.2	1.0	11.9
Erythema infectiosum	1.7	3.1	6.6	1.2	8.9
Exanthema subtium	0.7	0.5	0.3	1.8	2.7
Pertussis	0.3	0.4	0.1	0.8	2.1
Rubella	0.1	0.1	0.0	1.7	19.6
Herpangina	7.8	6.2	6.7	4.2	6.7
Measles	1.2	3.8	4.5	4.2	8.2
Mumps	2.0	5.9	11.2	3.4	9.5

Data of fiscal years are used in 3 years from 1999 through 2001.

The proportion of epidemic warning weeks: the number of epidemic warnings which were issued in all the public health center area during the fiscal year 1999-2001 divided by the number of weeks in all the public health center area (583 areas) during the fiscal year 1999-2001(157 weeks).

The temporal changes in the number of cases per week per sentinel medical institution before and after a warning onset are summarized in Table 4. For influenza-like illness, the median index of weeks in which the 596 epidemic warnings were issued in 1999-2001 was 37.0. The index increased markedly in the 4 weeks before the warnings were made as seen by following changes in median values: 1.3 on the 4th week before the warnings had been issued, 3.5 on the 3rd week, 9.0 on the 2nd week, and 20.0 on the 1st week. The median index was observed to then decrease gradually, being 39.0 in the 1st week after the warning had been issued, 29.9 on the 2nd week, 18.7 on the 3rd week, and 11.3 on the 4th week. These median values were higher than those measured in other weeks. For hand-foot-mouth disease and herpangina, we also observed a rapid increase in the value of median indices prior to an epidemic warning and a gradual decrease after this warning similar to that seen with influenza-like illness. However, for pertussis and rubella, these changes in the value of the median indices before and after epidemic warnings onset were not observed.

**Table 4.** Median, the 25th and 75th percentile of weekly cases per sentinel medical institution before and after the issuing of an epidemic warning.

		Weekly cases per sentinel medical institution									

		Before warning onset				Onset week	After warning onset				Non-warning weeks
	
		4weeks	3weeks	2weeks	1week		1week	2weeks	3weeks	4weeks	
Influenza-like illness	median	1.3	3.5	9.0	20.0	37.0	39.0	29.9	18.7	11.3	0.0
	25th percentile	0.3	1.2	5.2	14.6	32.5	28.2	20.3	11.9	5.8	0.0
	75th percentile	4.0	7.4	14.9	25.0	44.2	52.5	44.9	30.0	18.9	0.3
	number of observation	596	596	596	596	596	595	594	586	579	84,353

Pharyngoconjunctival fever	median	0.0	0.0	0.0	0.1	1.1	0.3	0.3	0.3	0.2	0.0
	25th percentile	0.0	0.0	0.0	0.0	1.0	0.0	0.0	0.0	0.0	0.0
	75th percentile	0.2	0.3	0.3	0.5	1.5	1.0	0.8	0.8	0.6	0.0
	number of observation	955	955	955	955	955	949	946	942	939	80,050

Group A streptococcal pharyngitis	median	1.3	1.5	1.9	2.0	4.5	2.8	2.3	2.3	2.0	0.4
	25th percentile	0.7	0.7	1.0	1.3	4.0	1.5	1.2	1.0	1.0	0.0
	75th percentile	2.0	2.3	2.7	3.0	5.3	4.0	4.0	3.6	3.8	1.0
	number of observation	873	873	873	873	873	870	867	862	854	81,355

Infectious gastroenteritis	median	6.3	8.1	11.1	15.0	23.0	21.7	18.5	14.6	12.3	2.7
	25th percentile	3.7	5.3	8.0	12.2	21.0	16.3	12.5	9.0	8.0	0.9
	75th percentile	10.0	11.6	14.0	17.5	26.3	28.3	27.4	21.8	18.3	5.8
	number of observation	1,012	1,012	1,012	1,012	1,012	1,011	1,010	1,004	996	79,140

Chickenpox	median	2.7	3.0	4.3	3.3	8.0	4.0	5.5	4.0	4.3	1.0
	25th percentile	1.5	1.5	3.0	2.0	7.3	2.7	3.6	2.3	2.5	0.3
	75th percentile	4.0	4.4	5.5	4.8	9.0	5.8	8.0	5.8	6.5	2.0
	number of observation	628	628	628	628	628	624	620	617	613	84,541

Hand-foot-mouth disease	median	0.8	1.3	2.0	3.0	6.0	6.1	6.1	5.3	4.5	0.0
	25th percentile	0.2	0.5	1.0	2.0	5.3	3.7	3.0	2.3	2.0	0.0
	75th percentile	1.7	2.1	3.0	4.0	7.5	8.6	10.2	10.4	10.0	0.4
	number of observation	743	743	743	743	743	743	743	742	741	80,735

Erythema infectiosum	median	0.5	0.5	0.7	0.8	2.3	1.1	1.0	1.0	0.8	0.0
	25th percentile	0.0	0.0	0.2	0.3	2.0	0.5	0.4	0.3	0.3	0.0
	75th percentile	1.0	1.0	1.1	1.3	2.7	2.0	2.0	1.8	1.6	0.3
	number of observation	611	611	611	611	611	609	604	598	593	84,750

Exanthema subtium	median	1.3	1.3	1.3	1.3	4.3	2.0	2.0	1.8	1.7	0.6
	25th percentile	0.9	1.0	0.7	1.0	4.0	1.0	1.0	1.0	0.8	0.3
	75th percentile	2.3	2.0	2.0	2.5	5.0	3.0	2.7	3.0	2.7	1.0
	number of observation	133	133	133	133	133	132	131	131	131	90,158

Pertussis	median	0.0	0.0	0.0	0.0	1.0	0.0	0.0	0.0	0.0	0.0
	25th percentile	0.0	0.0	0.0	0.0	1.0	0.0	0.0	0.0	0.0	0.0
	75th percentile	0.0	0.0	0.0	0.0	1.3	0.2	0.0	0.0	0.0	0.0
	number of observation	134	134	134	134	134	133	133	133	133	90,271

Rubella	median	0.0	0.0	0.0	0.0	3.5	0.0	0.0	0.0	0.0	0.0
	25th percentile	0.0	0.0	0.0	0.0	3.0	0.0	0.0	0.0	0.0	0.0
	75th percentile	0.3	0.8	0.3	0.7	5.0	2.0	2.0	1.0	0.0	0.0
	number of observation	11	11	11	11	11	11	11	11	11	91,409

Herpangina	median	0.5	1.0	2.2	3.8	7.5	8.0	7.8	6.0	4.3	0.0
	25th percentile	0.1	0.5	1.0	2.5	6.5	5.6	4.6	3.3	2.2	0.0
	75th percentile	1.3	2.0	3.3	4.9	9.2	11.3	12.0	10.5	7.7	0.3
	number of observation	972	972	972	972	972	972	972	972	972	77,651

Measles	median	0.1	0.2	0.4	0.4	1.9	0.8	0.8	0.6	0.6	0.0
	25th percentile	0.0	0.0	0.0	0.0	1.6	0.1	0.0	0.0	0.0	0.0
	75th percentile	0.5	0.5	0.8	0.8	2.3	1.4	1.6	1.5	1.3	0.0
	number of observation	449	449	449	449	449	444	442	442	440	85,802

Mumps	median	1.5	2.5	2.7	2.5	5.7	3.0	3.7	4.0	3.0	0.5
	25th percentile	1.0	1.7	1.7	1.5	5.3	2.0	2.5	2.5	1.8	0.0
	75th percentile	2.7	3.5	3.6	3.4	6.5	4.5	5.0	5.7	4.8	1.0
	number of observation	504	504	504	504	504	495	487	480	475	83,647

The data are expressed as median, the 25th and 75th percentile of cases per week per sentinel medical institution.

The number of observations represents the total number of weeks in which indices fall into the various categories.

The period from the beginning of week in April 1999 till the end of week in March 2002 are used for the analysis.

The proportions of weeks over the period of 1999-2001 in which a pre-epidemic warning was made for influenza-like illness and the 4 pediatric diseases, chickenpox, rubella, measles, and mumps in the 583 public health center areas is shown in Table 5. For influenza-like illness, the proportions of weeks with a pre-epidemic warning were 5.7%, 2.7%, and 4.6% in 1999, 2000, and 2001, respectively. These proportions were similar to those recorded in 1993-1997. For rubella, the proportion of weeks with a pre-warning warning in 1999-2001 was lower than in 1993-1997.

**Table 5.** The proportions of weeks in which an pre-epidemic warnings were issued in public health center areas from April 1999 through March 2002, compared with the range of those in 1993-1997.

The proportion of pre-epidemic warning weeks (%)					

Diseases	April 1999-March 2000	April 2000-March 2001	April 2001-March 2002	Range(1993-1997)	

				minimum	maximum
Influenza-like illness	5.7	2.7	4.6	1.9	4.5
Chickenpox	6.0	7.4	5.8	6.2	6.9
Rubella	0.4	0.2	0.2	3.4	9.5
Measles	2.3	6.2	5.8	5.2	8.2
Mumps	1.9	4.0	6.0	2.1	4.7

Data of fiscal years are used in 3 years from 1999 through 2001.

The proportion of pre-epidemic warning weeks: the number of pre-epidemic warnings which were issued in all the public health center area during the fiscal year 1999-2001 divided by the number of weeks in all the public health center area (583 areas) during the fiscal year 1999-2001(157 weeks).

Table 6 shows the temporal changes in the number of cases per week per sentinel medical institution before and after a pre-epidemic warning onset. For influenza-like illness, the median index in the 1,333 in which a pre-epidemic warning was issued over the period of 1999-2001 was 13.6. The median index increased in the 4 weeks prior to this warning and was found to be 0.5 on the 4th week before the pre-warning had been issued, 1.1 on the 3rd week, 2.8 on the 2nd week, and 6.3 on the 1st week. The median index continued to increase and then decreased gradually being 19.0, 20.7, 18.0, and 12.7 on the 1st, 2nd, 3rd, and 4th weeks after the warning, respectively. These median indices were higher than those measured in the other weeks. For chickenpox and mumps, the median indices before and after the pre-epidemic warning were also higher than in the other weeks, whereas for rubella and measles, many of the indices before and after the warning were zero, similar to that observed in the other weeks.

**Table 6.** Median, the 25th and 75th percentile of weekly cases per sentinel medical institution before and after the issuing of a pre-epidemic warning.

		Weekly cases per sentinel medical institution									

						Onset week					Other weeks*
	
		4weeks	3weeks	2weeks	1week		1week	2weeks	3weeks	4weeks	
Influenza-like illness	median	0.5	1.1	2.8	6.3	13.6	19.0	20.7	18.0	12.7	0.0
	25th percentile	0.0	0.3	1.3	4.3	11.5	12.6	12.3	8.9	5.9	0.0
	75th percentile	1.4	2.6	4.8	8.2	17.1	27.4	32.5	30.0	23.0	0.1
	number of observation	1,333	1,333	1,333	1,333	1,333	1,332	1,325	1,317	1,308	79,626

Chickenpox	median	2.0	2.0	2.5	2.0	4.5	2.7	3.4	2.7	2.9	0.9
	25th percentile	1.0	1.0	1.7	1.3	4.1	1.7	2.3	1.7	1.8	0.3
	75th percentile	3.0	3.0	3.3	3.0	5.1	3.8	4.9	4.0	4.3	1.8
	number of observation	2,239	2,239	2,239	2,239	2,239	2,220	2,205	2,191	2,184	72,190

Rubella	median	0.0	0.0	0.0	0.0	1.0	0.0	0.0	0.0	0.0	0.0
	25th percentile	0.0	0.0	0.0	0.0	1.0	0.0	0.0	0.0	0.0	0.0
	75th percentile	0.0	0.0	0.0	0.0	1.5	0.3	0.2	0.0	0.0	0.0
	number of observation	154	154	154	154	154	151	150	149	148	90,185

Measles	median	0.0	0.0	0.0	0.0	0.6	0.2	0.2	0.0	0.0	0.0
	25th percentile	0.0	0.0	0.0	0.0	0.5	0.0	0.0	0.0	0.0	0.0
	75th percentile	0.2	0.2	0.3	0.2	0.8	0.5	0.5	0.4	0.5	0.0
	number of observation	1,818	1,818	1,818	1,818	1,818	1,803	1,787	1,779	1,772	75,926

Mumps	median	1.5	1.7	1.7	1.5	3.3	2.0	2.0	2.3	2.0	0.4
	25th percentile	0.8	1.0	1.0	1.0	3.0	1.1	1.3	1.5	1.0	0.0
	75th percentile	2.1	2.3	2.3	2.0	3.8	2.8	3.0	3.3	3.0	1.0
	number of observation	1,444	1,444	1,444	1,444	1,444	1,435	1,421	1,408	1,395	79,159

The data are expressed as median, the 25th and 75th percentile of cases per week per sentinel medical institution.

The number of observations represents the total number of weeks in which indices fall into the various categories.

The period from the beginning of week in April 1999 till the end of week in March 2002 are used for the analysis.

*Other weeks: weeks that exclude the week of onset and before and after the onset of a pre-epidemic warning.

The sensitivity, specificity, and positive predictive values of the pre-epidemic warnings are listed in Table 7. For influenza-like illness, the sensitivity was 90.4%; the specificity was 93.7% and the positive predictive value 23.9%. For the four pediatric diseases, the sensitivity ranged between 25.1% and 54.2%, the specificity between 86.1% and 99.2%, and the positive predictive value between 2.5 and 20.8%.

**Table 7.** The sensitivity, specificity, and positive predictive values of an pre-epidemic warning in infectious disease surveillance in Japan, April 1999-March 2002.

	Sensitivity	Specificity	Positive predictive value
Influenza-like illness	90.4	93.7	23.9
Chickenpox	54.2	86.1	17.7
Rubella	36.4	99.2	2.5
Measles	43.7	89.3	16.3
Mumps	25.1	91.7	20.8

The numbers in the table are expressed as percentage (%)

The above result is based on the analysis of all the weekly cases per sentinel medical institution in the public health center areas (583 areas), from the beginning of week in April 1999 till the end of week in March 2002 (157 weeks).

The sensitivity is the proportion of valid pre-warnings, in the 4 weeks before the onset of an epidemic warning.

The specificity is the proportion of weeks without a pre-epidemic warning relative to the total number of weeks, in which there was no epidemic warning nor the 4 weeks before and after epidemic warning.

The positive predictive value is the proportion of valid pre-epidemic warnings relative to the total of pre-epidemic warnings.

## DISCUSSION

This study is to evaluate a method of pre-epidemic and epidemic warning by examining a huge number of observations. For this purpose, we decided to provide a detailed description of data and not to use a method of statistical testing.

In this study, the critical values for determining when epidemic warnings should be issued at both the onset and the end of an epidemic were determined using data of the frequency of epidemic warnings from 1993 through 1997. The reason for establishing these critical values is that the definition of epidemic is not well standardized in many infectious diseases, and also that neither frequent nor very rare warnings are adequate for public health standards. In many of the diseases we investigated, including influenza-like illness, the proportion of weeks in which an epidemic warning was issued was very similar in the two study periods. With regard to the frequency of epidemic warnings, the method for determining when an epidemic warning should be issued in the current surveillance system was as applicable as that in the previous surveillance system. However, the frequency of epidemics of exanthema subtium, pertussis, and rubella during a 3 year period from 1999 through 2001 was lower than that in 1993-1997. With regard to rubella, the number of cases per week per sentinel medical institution in 1999-2001 was considerably lower than in 1993-1997, a difference that was reflected by a lower prevalence of epidemics in 1999-2001. For exanthema subtium and pertussis, the patterns of epidemics appeared to be different between the two periods and therefore it may be necessary to modify the critical values of these epidemic warning.

In a situation when many cases occur in the weeks immediately after an epidemic warning, such warnings are useful because they initiate public health activities against the epidemic. In our study we examined the temporal changes in the numbers of cases per week per sentinel medical institution before and after an epidemic warning over the period of 1999-2001. For several diseases including influenza-like illness, we observed a gradual decrease in the index after the onset of an epidemic warning. This observation suggested that an epidemic warning may in itself result in useful public health activities against an epidemic. However, such a desirable temporal change was not observed for diseases such as pertussis and rubella.

The frequency of pre-epidemic warnings for influenza-like illness, chickenpox, measles, and mumps in 1999-2001 was similar to that recorded in 1993-1997. With regard to the frequency of pre-epidemic warnings, the method used currently for issuing these warnings was as applicable as that used in previous surveillance system. For example, although the frequency of pre-epidemic warnings for rubella in 1999-2001 was lower than in 1993-1997 this may simply reflect that there was a lower incidence of this disease in 1999-2001.

In this study we also examined the temporal changes in the number of cases per week per sentinel medical institution before and after a pre-epidemic warning in 1999-2001. For influenza-like illness, the index continued to increase and then decreased gradually after the warning had been issued. This pattern of temporal changes is a desirable outcome of a pre-epidemic warning. In contrast, pre-epidemic warnings for the four other diseases we investigated did not result in these desirable temporal changes.

The sensitivity, specificity, and positive predictive value of the critical values selected for issuing a pre-epidemic warning were also determined using surveillance data in 1993-1997. The specificity of the pre-epidemic warnings issued in 1999-2001 for all the diseases investigated ranged between 86.1% and 99.2%. We have pointed out in a previous report that pre-epidemic warnings require a high specificity for routine infectious disease surveillance. The results of the present study confirm that pre-epidemic warnings in 1999-2001 had suitably high specificity. Our data also showed the sensitivity of the critical values was 90.4% in influenza-like illness and between 25.1% and 54.2% in other diseases. As discussed above, there may have been only the small number of rubella cases, and as we have pointed out previously, it would not be expected to find high positive predictive value when epidemics were not frequent. Overall these results indicate that pre-epidemic warnings in 1999-2001 for influenza-like illness had sufficient sensitivity, but that detection of other four diseases at an early stage of an epidemic may not be easy. We showed the efficiency of pre-warning on influenza-like illness and extra years of observation is needed in the further examination of the other four diseases.

Our study had some inherent problems and limitations. The major problem was the definition of an epidemic with warnings being issued on the basis of the number of cases per week per sentinel medical institution. It is possible that the critical value used may vary according to the type of disease, season, and area. Although 3 years’ data were available after the introduction of the expanded surveillance system, additional data are necessary in order to examine variation in the critical values.

In summary, this study examined the frequency of epidemic and pre-epidemic warnings, the temporal changes in the number of cases per week per sentinel medical institution before and after these warnings, and the sensitivity, specificity and positive predictive values of pre-epidemic warnings. We suggest other view-points (e.g. early detection of epidemic dispersion) may also be important for evaluating epidemic and pre-epidemic warnings.
